# Parcellation influence on the connectivity‐based structure–function relationship in the human brain

**DOI:** 10.1002/hbm.24866

**Published:** 2019-11-19

**Authors:** Arnaud Messé

**Affiliations:** ^1^ Department of Computational Neuroscience University Medical Center Eppendorf, Hamburg University Hamburg Germany

**Keywords:** brain connectivity, parcellation, structure–function relationship

## Abstract

One of the fundamental questions in neuroscience is how brain structure and function are intertwined. MRI‐based studies have demonstrated a close relationship between the physical wiring of the brain (structural connectivity) and the associated patterns of synchronization (functional connectivity). However, little is known about the spatial consistency of such a relationship and notably its potential dependence on brain parcellations. In the present study, we performed a comparison of a set of state‐of‐the‐art group‐wise brain atlases, with various spatial resolutions, to relate structural and functional connectivity derived from high quality MRI data. We aim to investigate if the definition of brain areas influences the relationship between structural and functional connectivity. We observed that there is a significant effect of brain parcellations, which is mainly driven by the number of areas; there are mixed differences in the SC–FC relationship when compared to purely random parcellations; the influence of the number of areas cannot be attributed solely to the reliability of the connectivity estimates; and beyond the influence of the number of regions, the spatial embedding of the brain (distance effect) can explain a large portion of the observed relationship. As such the choice of a brain parcellation for connectivity analyses remains most likely a matter of convenience.

## 1 INTRODUCTION

One of the fundamental questions in neuroscience is how brain structure and function are intertwined. In that context, thanks to lightning technological and analytical progress and its noninvasiveness, MRI has upset the field by highlighting the architecture of structural connections and of functional interactions of the brain. Pioneer studies have demonstrated a tight intricate association between the patterns of structural and functional connectivity (Greicius, Supekar, Menon, & Dougherty, 2009; Hagmann et al., 2008; Koch, Norris, & Hund‐Georgiadis, 2002; Vincent et al., 2007). However, it remains unclear whether this relationship is spatially consistent or if it depends on brain parcellation or atlas.

In contrast to other organs, the brain has a peculiar topographic organization. This organization has led to the concept of brain area or region (Amunts & Zilles, 2015; Eickhoff, Constable, & Yeo, 2017). Beyond theoretical aspects, the concept of brain area has practical considerations, allowing a meaningful reduction in dimension and noise. To date, there exists a multitude of definitions of what constitute a brain region, each featuring specific aspects of the brain. Brain regions can be defined by microstructural properties including cyto‐ or myeloarchitectonic information (Brodmann, 1909; Von Economo & Koskinas, 1925; Zilles, Palomero‐Gallagher, & Amunts, 2015a, 2015b), as well as by more global macroscopic features, such as connectivity patterns (Passingham, Stephan, & Kotter, 2002). Consequently, numerous connectivity‐based parcellations from MRI data have been proposed (e.g., Fan et al., 2016; Glasser et al., 2016; Gordon et al., 2016; Joliot et al., 2015; Power et al., 2011; Shen, Tokoglu, Papademetris, & Constable, 2013; Thomas Yeo et al., 2011), with a large range of granularity and ambiguous overlap between methods (Eickhoff et al., 2017). Little is known about the potential consequences of such a variety in terms of subsequent connectivity analysis.

In the present study, we performed a comparison of a set of state‐of‐the‐art group‐wise brain atlases to relate structural and functional connectivity (SC and FC, respectively) derived from high quality MRI data. The set of brain atlases comprises various levels of assumption and spatial resolution (i.e., number of regions). We aim to investigate if the definition of brain areas influences the relationship between SC and FC and to establish the potential factors driving it. One of the main influential factors in brain connectivity analysis is the size of the areas, and, consequently their number. While fine‐grained areas may be prone to low signal‐to‐noise ratio and hence a lack of reliable connectivity estimates, large areas may overlook some details by averaging out disparate signals (Stanley et al., 2013). The SC–FC relationship was quantified via the use of three main criteria: *SC–FC correlation* (i.e., the correlation between the patterns of SC and FC), *SC–FC partition* (i.e., the overlap between communities extracted from SC and FC), and *SC–FC fingerprint* (i.e., the proportion of subjects who can be identified from their SC–FC correlation).

We observed that (a) there is a significant effect of brain parcellations on the SC–FC correlation and fingerprint, which is mainly driven by the number of areas; (b) the overlap between SC and FC partitions is virtually not influenced by the choice of the atlas nor by any normalization; (c) there are mixed differences in the SC–FC relationship when compared to purely random parcellations; (d) the influence of the number of areas cannot be attributed solely to the reliability of the connectivity estimates; (e) and beyond the influence of the number of regions, the spatial embedding of the brain (distance effect) can explain a large portion of the observed relationship.

## 2 MATERIALS AND METHODS

### 2.1 HCP data

The study included MRI data from the publicly available Human Connectome Project repository (HCP; http://www.humanconnectome.org/; Van Essen, Uǧurbil, et al., 2012). The scanning protocol was approved by the Washington University in St. Louis's Human Research Protection Office. Data for a total of 94 young adults (age: 29.1 ± 3.5 years; 45 females) of the HCP S1200 public data release with complete diffusion MRI (dMRI), resting‐state functional MRI (rfMRI), and MEG data were included in this study. We investigated functional connectivity extracted from resting‐state fMRI acquisitions. All subjects provided written informed consent and were scanned on a 32‐channel 3T Siemens Skyra scanner (Siemens, Erlangen, Germany).

Resting‐state functional MRI images were acquired while the participants relaxed with eyes open using a gradient‐echo EPI sequence with multiband factor 8, time repetition (TR) 720 ms, time echo (TE) 33.1 ms, flip angle 52°, 104 × 90 matrix size, 72 slices, 2 mm isotropic voxels, and 1200 time points. Scans were repeated twice using different phase‐encoding directions (RL and LR) during 2 sessions (REST1 and REST2). Diffusion MRI data were recorded using a spin‐echo EPI sequence with multiband factor of 3, TR 5.52 s, TE 89.5 ms, flip angle 78°, 168 × 144 matrix size, 111 slices, 1.25 mm isotropic voxels. Two hundred and seventy encoding directions distributed over three diffusion shells of b‐values 1000, 2000, and 3000 s/mm^2^ and 18 non‐weighted images (*b* = 0) were acquired for each subject. A structural 3D MPRAGE T1‐weighted volume was also acquired (TR: 2.4 s, TE: 2.14 ms, TI: 1000 ms, flip angle 8°, 320 × 320 matrix size, voxel size: 0.7 mm isotropic). For further details about the MRI protocols see Moeller et al., 2010; Feinberg et al., 2010; Setsompop et al., 2012; Sotiropoulos et al., 2013; Smith et al., 2013; Uǧurbil et al., 2013.

We used the “minimally preprocessed” dataset together with the denoised rfMRI data. The minimal preprocessing pipelines include spatial preprocessing: artifact and gradient distortion correction, field map processing, subcortical segmentation, surface generation (including pial and gray‐white matter boundary), cross‐modal registration, and spatial normalization to the Montreal Neurological Institute standard space; as well as dedicated preprocessing steps for rfMRI and dMRI data. All data were mapped to a standard gray ordinates space, composed of a set of gray matter cortical vertices and subcortical voxels (≈2 mm spacing), providing a one‐to‐one correspondence between individuals (Glasser et al., 2013). The rfMRI data were smoothed with a 2 mm FWHM Gaussian kernel, high‐pass filtered (1/2000 Hz frequency cut‐off) and denoised using FIX a method based on independent component analysis (Salimi‐Khorshidi et al., 2014; Smith et al., 2013). Fiber orientations density function from the multi‐shell dMRI data were inferred using the ball & stick model implemented in FSL (Behrens, Johansen‐Berg, Jbabdi, Rushworth, & Woolrich, 2007; Jbabdi, Sotiropoulos, Savio, Graña, & Behrens, 2012). The parameters included up to 3 fibers per voxel.

### 2.2 Brain parcellations

We used a set of 24 publicly available state‐of‐the‐art group‐wise cortical brain parcellations recently investigated in Arslan et al., 2018. Among these atlases, 10 were pre‐computed and retrieved from the literature, and the remaining were computed from rfMRI acquisitions of 100 unrelated HCP subjects in Arslan et al., 2018 and possessed multiple levels of spatial resolution (i.e., number of regions).^1^ These brain atlases are defined on the standard cortical surfaces and freely available (https://biomedia.doc.ic.ac.uk/brain-parcellation-survey/). Furthermore, we added subcortical gray matter structures provided by the minimal preprocessing pipelines, including the thalamus, caudate, putamen, pallidum, hippocampus, amygdala, accumbens, diencephalon ventral, and the cerebellum, all bilaterally. In total, 144 state‐of‐the‐art group‐wise cortical brain parcellations were used.

We also generated spatially random cortical parcellations based on a Poisson disc sampling (Arslan et al., 2018; Schirmer, 2015). For each state‐of‐the‐art parcellation, we generated 20 surrogate random parcellations with the same number of regions. First, a set of vertices equal to the number of regions are selected using an approximate blue noise sampling using the Geometry Processing Toolbox (Jacobson et al., 2018), defining regions center. Subsequently, each remaining vertex is assigned to its closest region center. These random parcellations were used as a null model to test the spatial significance of the proposed atlases.

Additionally, we generated random cortical parcellations with a fixed number of points sampled per area. For each state‐of‐the‐art parcellation, we generated 20 surrogate random parcellations with the same number of regions, where for each area we sampled uniformly a fixed number of vertices. The number of vertices sampled per area was fixed to 60, areas of size below this value (which represents less than 4% of all areas) were kept as such. These random parcellations with a fixed number of sampled points were used as a null model to test the SC and FC reliability and its potential effect on the subsequent SC–FC relationship across atlases.

The composition of brain atlases was compared using the adjusted Rand index (Hubert & Arabie, 1985). The Rand index quantifies the similarity between two partitions or atlases of the brain by computing the proportion of points (vertices or voxels) for which the two partitions are consistent (i.e., they are either in the same area or in a different area for both partitions). The adjustment accounts for the level of similarity that would be expected by chance only.

### 2.3 Connectivity measures

#### 2.3.1 Structural connectivity

We used the probabilistic white matter tracking method implemented in FSL (Behrens et al., 2007) to track all possible connections between all pairs of gray matter points (either vertices or voxels) in the “matrix3” mode. For every voxel of the white matter, we initiated 5,000 samples. Starting points were chosen randomly within a sphere of radius 2 mm from the voxel's center. Initial orientation was randomly chosen and then streamlines were grown in the two opposite directions with a propagation step set at 0.5 mm and a maximal curvature at 80°.

Fiber tracking was stopped when samples reached the gray‐white matter boundary surface, the subcortical gray matter structures or the ventricles. Samples reaching the ventricles were rejected. Samples were kept only if they reached gray‐white matter boundary surface and/or subcortical gray matter structures in the two opposite directions. SC was then defined as the total number of samples connecting pairs of regions. Likewise, we built a matrix of the average fibers' length between regions.

We also investigated the potential influence of candidate normalization procedures. First, we computed one of the most commonly used approaches (*surface normalization*), which consists of expressing SC as a density of samples per unit surface, by dividing the number of samples by the average surface area of the regions (Hagmann et al., 2008). Second, we used a normalization inspired by tract‐tracing studies (*fractional scaling*), which consists of computing the fraction of samples relative to the total number of samples that reach each region excluding self (within region) connections (Donahue et al., 2016).

Additionally, we also mitigated the distance‐related bias towards longer fibers inherent from tractography algorithms. SC was further either weighted (multiplied) or regressed by the average fiber length. As SC is biased by distance in a logarithmic way (Roberts, Perry, Roberts, Mitchell, & Breakspear, 2017) (Figure S1), we regressed out the distance on the logarithm of SC and then took the power of the residuals as the corrected SC.

#### 2.3.2 Functional connectivity

Functional connectivity was computed from resting‐state fMRI acquisitions. For each subject, phase‐encoding direction, session and atlas, the time series of all vertices or voxels within a given brain region was spatially averaged to form the representative BOLD signal of that region. FC was then computed as the Pearson correlation between all possible pairs of brain region time series. To obtain robust estimates, FC matrices were averaged across runs (phase‐encoding directions and sessions).

We also investigated the potential influence of spurious sources of variance, by regressing out linear and quadratic drifts, motion parameters, and the global brain signal (Fox, Zhang, Snyder, & Raichle, 2009; Van Dijk et al., 2010).

#### 2.3.3 Summary

For each subject and brain atlas (state‐of‐the‐art or randomly generated), the preprocessing yielded 12 matrices: 9 of SC (3 normalizations × 3 distance‐corrections), 2 of FC (with or without regression), and one of average fibers' length. The reference set of connectivity matrices corresponds to SC without any normalization or distance correction (raw number of samples), and FC without regression of spurious sources of variance.

### 2.4 Connectivity‐based structure–function relationship

**Figure 1:**
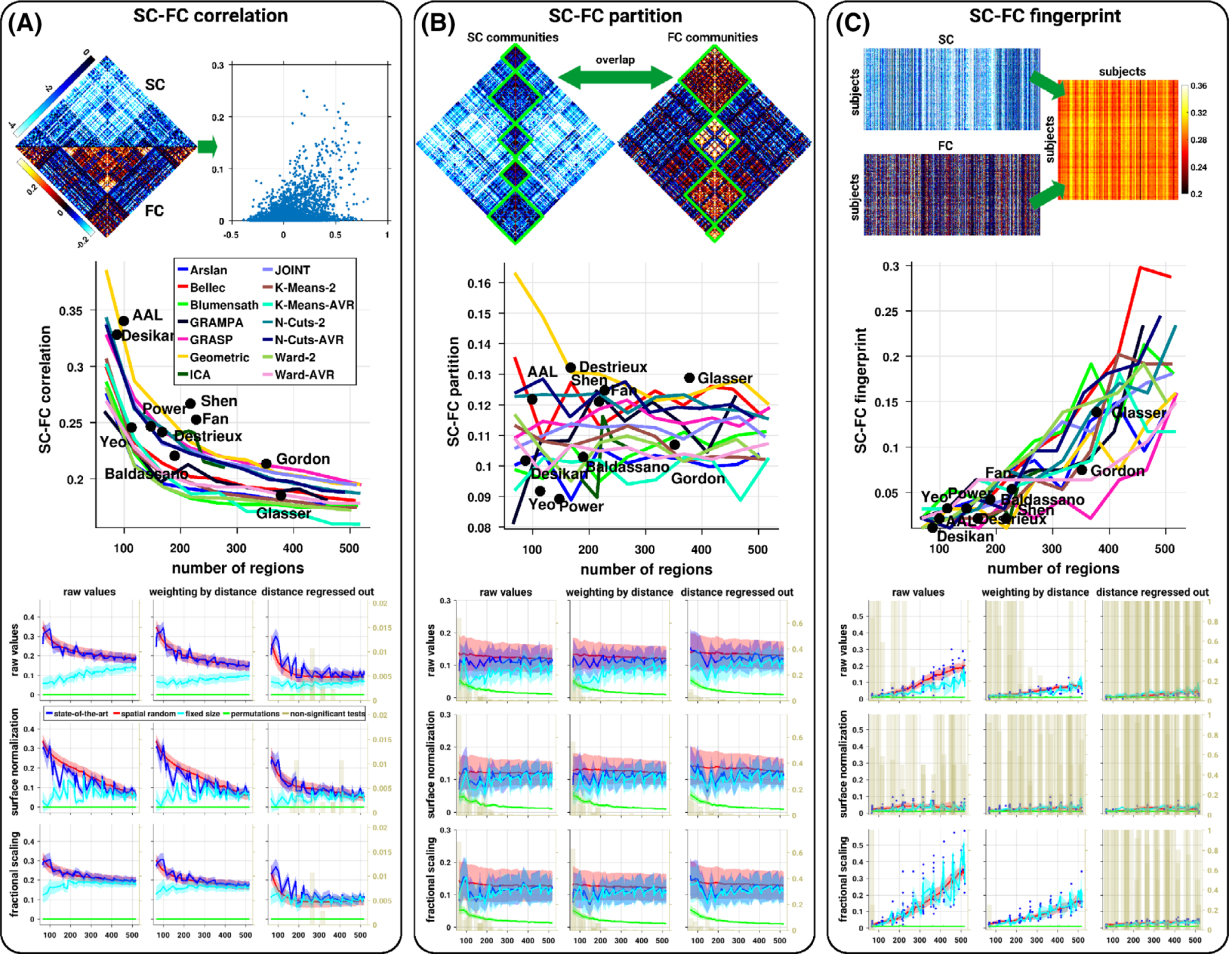
Brain SC–FC relationship across atlases. (A) SC–FC correlation, that is, the correlation between structural and functional connectivity patterns, computed for each subject and each atlas. (B) SC–FC partition, that is, the overlap between communities extracted from SC and those from FC, computed for each subject and each atlas. (C) SC–FC fingerprint, that is, the proportion of subjects correctly identified according to their SC–FC correlation. Across the panels, the first row represents an illustration of the SC–FC relationship measures, the second row represents the average values per atlas as a function of the number of regions, the last row represents the SC–FC relationship according to various SC normalizations (subplots) as a function of the number of regions. For each subplot, the blue, red, cyan, and green curves represent the mean and *SD* (shaded area) across subjects and atlases of the same size of the SC–FC relationship from the state‐of‐the‐art atlases, spatially random parcellations, fixed size parcellations, and permutations, respectively. The blue dots in the SC–FC fingerprint subplots represent the state‐of‐the‐art atlases. The dark beige histograms represent the proportion of subjects (or the proportion of atlases for the SC–FC fingerprint) at a given number of regions for whom there is no significant statistical difference compared to random expectations (permutation‐based tests corrected, *p* < .01)

The relationship between structural and functional connectivity was assessed both in terms of strength similarity (*SC–FC correlation*), overlap in partitions (*SC–FC partition*), and connectivity fingerprint (*SC–FC fingerprint*; see Figure 1). Strength similarity was evaluated by means of the Pearson correlation between the patterns of SC and FC. Overlap between partitions was investigated based on the normalized mutual information between the communities detected in SC and those in FC. Communities were extracted by means of a greedy optimization method that attempts to maximize a measure of network modularity, the Louvain algorithm (Blondel, Guillaume, Lambiotte, & Lefebvre, 2008). We used an asymmetric generalization of the measure of network modularity adapted for fully connected network with signed values (Rubinov & Sporns, 2011). Community detection was performed using the function *community_louvain.m* as provided by the Brain Connectivity Toolbox (http://www.brain-connectivity-toolbox.net/) with the default resolution parameter value (*γ* = 1). To limit the computational burden, the detection was performed on only one run per subject and atlas. Connectivity fingerprint were evaluated by computing the proportion of subjects who can be identified from their SC–FC correlation, similar to the approach developed in Finn et al. (2015) and Zimmermann, Griffiths, Schirner, Ritter, & McIntosh (2019). For each individual subject, we computed the correlation between its own FC and the SCs of all subjects (including itself), to find the pair of maximally similar SC and FC matrices.

Of note, the SC–FC correlation and SC–FC partition are defined at the subject level while the SC–FC fingerprint is defined at the group level.

### 2.5 Statistical assessments

In order to test the significance of the SC–FC relationship we used three null models:

- The first null model was based on permutations, the Mantel test (Mantel, 1967), where for each subject and each atlas, we generated 1000 surrogate SC matrices (after any potential SC normalization or correction) in which the region's connectivity profiles were preserved, but brain regions were randomly permuted. This procedure tests the significance of the SC–FC relationship against random expectations.
- The second null model was based on spatially random parcellations described above, where for each state‐of‐the‐art atlas, we generated 20 surrogate parcellations and their associated connectivity matrices. This procedure tests the significance of the SC–FC relationship in the state‐of‐the‐art brain atlases against spatially random expectations. We normalized the SC–FC relationship measures to that of the corresponding random parcellations using a “*z*‐score”‐like approach (subtracting and dividing individual values per subject and per atlas by the mean and *SD* obtained from the set of associated random parcellations). We then used *z*‐tests to measure the significance of the observed differences.
- The third null model was based on random parcellations with a fixed number of points sampled per area described above, where for each state‐of‐the‐art atlas, we sampled 20 surrogate parcellations and their associated connectivity matrices. This procedure tests the reliability of the SC and FC estimates and its subsequent effect on the SC–FC relationship. For each subject and each atlas, the reliability was estimated by computing the correlation between the SC (resp. FC) patterns extracted from the fixed size parcellations and the SC (resp. FC) pattern from the original state‐of‐the‐art atlas.

To test whether there were systematic differences in the SC–FC relationship measures between atlases, a paired Student's *t* test was performed between all possible pairs of atlases and over all the subjects, and we reported the Cohen's *d* effect size. Furthermore, to test whether relative differences between individuals were preserved across atlases, we computed the Pearson correlation between SC–FC relationship measures for each pair of brain atlases across subjects. Pairwise comparisons (either difference or correlation) were plotted as a function of the normalized absolute difference in the number of regions between the pair of atlases (i.e., absolute difference divided by the average).

All statistical tests were rejected at *p* < .01 significance. Correction for multiple comparisons was performed by controlling the false discovery rate when appropriate (Benjamini, Krieger, & Yekutieli, 2006).

## 3 RESULTS

Here using high quality MRI data, we explored the dependence of the relationship between structural and functional connectivity on brain parcellation. We used a total of 144 publicly available group‐wise whole‐brain atlases, and quantified the SC–FC relationship via the use of three main criteria: *SC–FC correlation* (i.e., the correlation between SC and FC patterns), *SC–FC partition* (i.e., the overlap between communities extracted from SC and FC), and *SC–FC fingerprint* (i.e., the proportion of subjects for whom SC and FC are the most similar). We would like to observe whether brain parcellation might have an effect on the resulting structure–function relationship.

### 3.1 Overall SC–FC relationship

Structure–function measures across atlases and subjects as a function of the spatial scale (i.e., number of regions) are represented in Figure 1. We observed general trends across measures, SC–FC correlation and SC–FC fingerprint were strongly influenced by the number of regions, with a global decrease (resp. increase) in correlation (resp. fingerprint) with increasing number of regions. There was no clear influence of the number of regions on the overlap between SC and FC partitions. Interestingly, the variability across subjects appeared to be small. SC–FC correlations were highly significant across all atlases, while for the SC–FC partition and the SC–FC fingerprint, only fine‐grained atlases (with a number of regions larger than ~200) had significant values (permutation‐based tests corrected).

### 3.2 What can be expected from spatially random parcellations?

**Figure 2:**
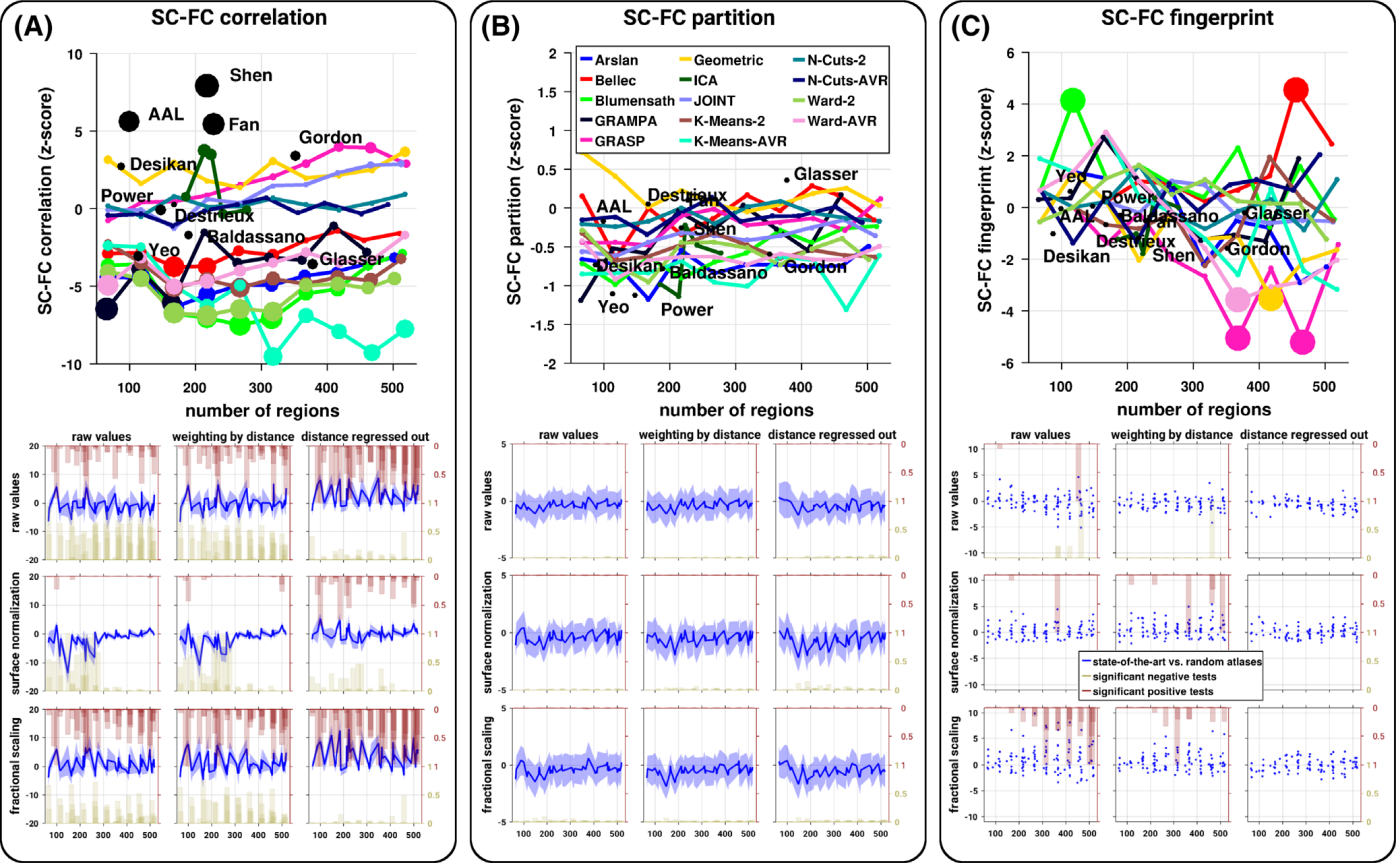
Brain SC–FC relationship, comparing state‐of‐the‐art atlases against spatially random parcellations. Normalized (*z*‐score‐like) SC–FC correlation (a), partitions overlap (b), and fingerprint (c). Across the panels, the first row represents the average normalized values per atlas as a function of the number of regions where the size of the points is proportional to the number of subjects significantly different from the random parcellations, the second row represents the normalized SC–FC relationship according to various SC normalizations (subplots) as a function of the number of regions. For each subplot, the blue curve represents the mean and *SD* (shaded area) of the normalized SC–FC relationship values across subjects and atlases of the same size of the state‐of‐the‐art atlases against random parcellations. The blue dots in the SC–FC fingerprint subplots represent the state‐of‐the‐art atlases. The dark beige (resp. red) histograms represent the proportion of subjects (or the proportion of atlases for the SC–FC fingerprint) at a given number of regions for whom there is a significant statistical negative (resp. positive) deviation from the random parcellations (*z*‐tests corrected, *p* < 0.01)

The atlases used here were based on a multitude of criteria; it is, however, unclear how their specificity impacts the resulting SC–FC relationship and whether they meaningfully deviate from purely spatially random surrogate parcellations. Surprisingly, we observed that the SC–FC relationship from the spatially random parcellations was quite close to the ones derived from the state‐of‐the‐art atlases (Figure 1). However, despite small differences, some of the state‐of‐the‐art atlases differed significantly from the random parcels (Figure 2). We observed a mixed proportion of subjects for whom there was a significant positive or negative deviation of the SC–FC correlation from the ones extracted from the random parcels. The overlaps between SC and FC partitions were not significantly different from the ones extracted from the random atlases, and only a small proportion of subjects showed a significant positive deviation in the SC–FC fingerprint (only for some atlases with a high number of regions).

### 3.3 On the reliability of connectivity estimates

**Figure 3:**
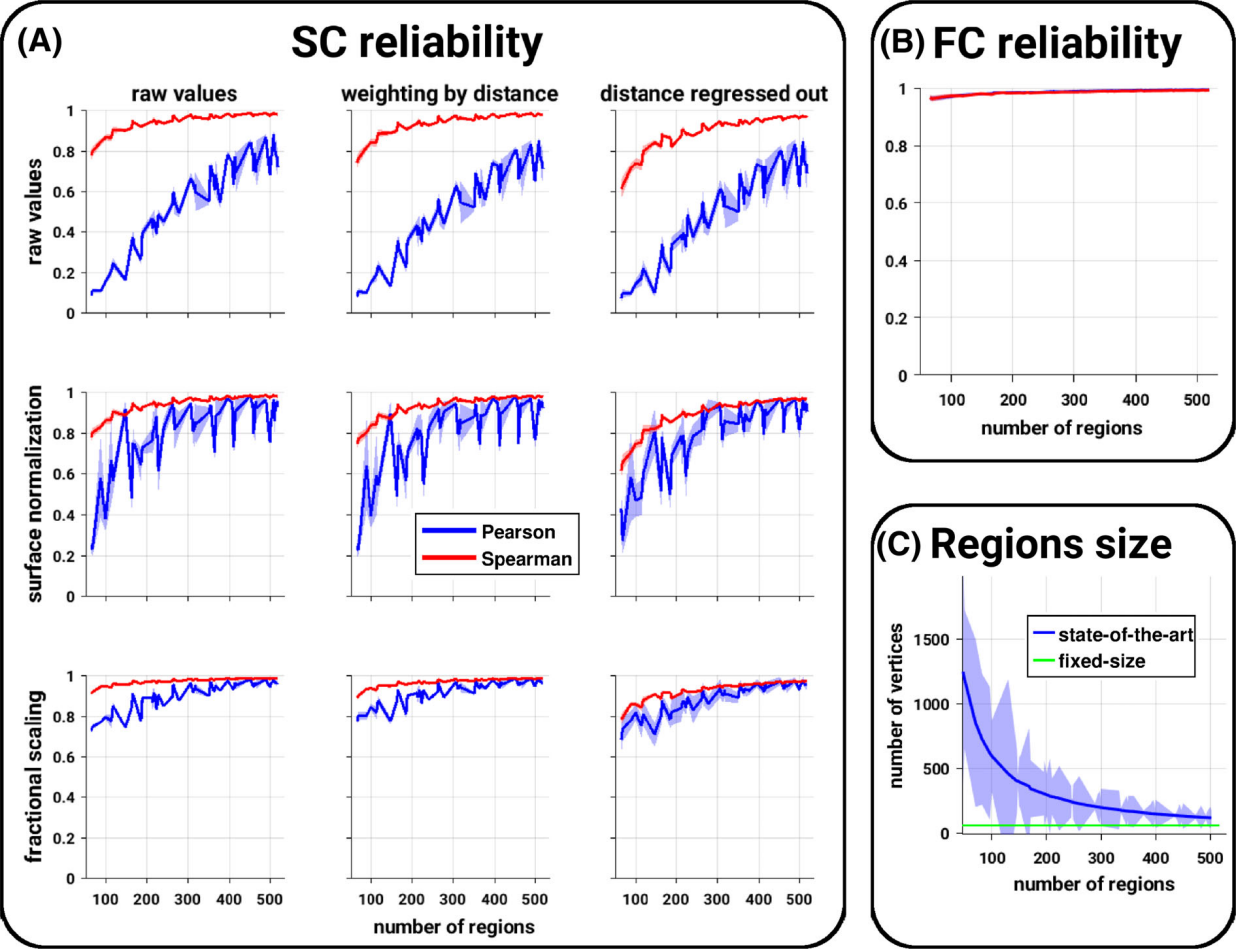
Reliability of connectivity estimates. (a) Structural connectivity reliability according to various normalizations (subplots) as a function of the number of regions. For each subplot, the blue (resp. red) curve represents the mean and *SD* (shaded area) across subjects and atlases of the same size of the Pearson (resp. Spearman) correlation between the structural connectivity estimates from the state‐of‐the‐art atlases and the parcellations of fixed size. (b) Functional connectivity reliability as a function of the number of regions. The blue (resp. red) curve represents the mean and *SD* (shaded area) across subjects and atlases of the same size of the Pearson (resp. Spearman) correlation between the functional connectivity estimates from the state‐of‐the‐art atlases and the parcellations of fixed size. (c) Size of the cortical regions as a function of the number of regions composing the atlases. The blue curve represents the mean and *SD* (shaded area) across regions and atlases of the same size of the number of vertices per regions. The green line represents the number of vertices of the fixed‐size atlases

The main effect observed in the SC–FC relationship is a general decrease in correlation as a function of the number of regions. One possible explanation is the reliability of the connectivity estimates, where coarser regions and hence atlases with low number of regions may have higher signal‐to‐noise ratio. We explored here such effect by fixing the number of sampled points per area. We observed that the reliability of SC estimates are strongly influenced by the size of the areas, while, surprisingly, FC estimates are virtually not affected (Figure 3).^2^ Consequently, while the resulting SC–FC partition and fingerprint were only slightly affected by the connectivity reliability, the relationship of the SC–FC correlation with the number of regions was inverted when computed using the fixed size constrain (Figure 1).

### 3.4 Pairwise comparisons

**Figure 4:**
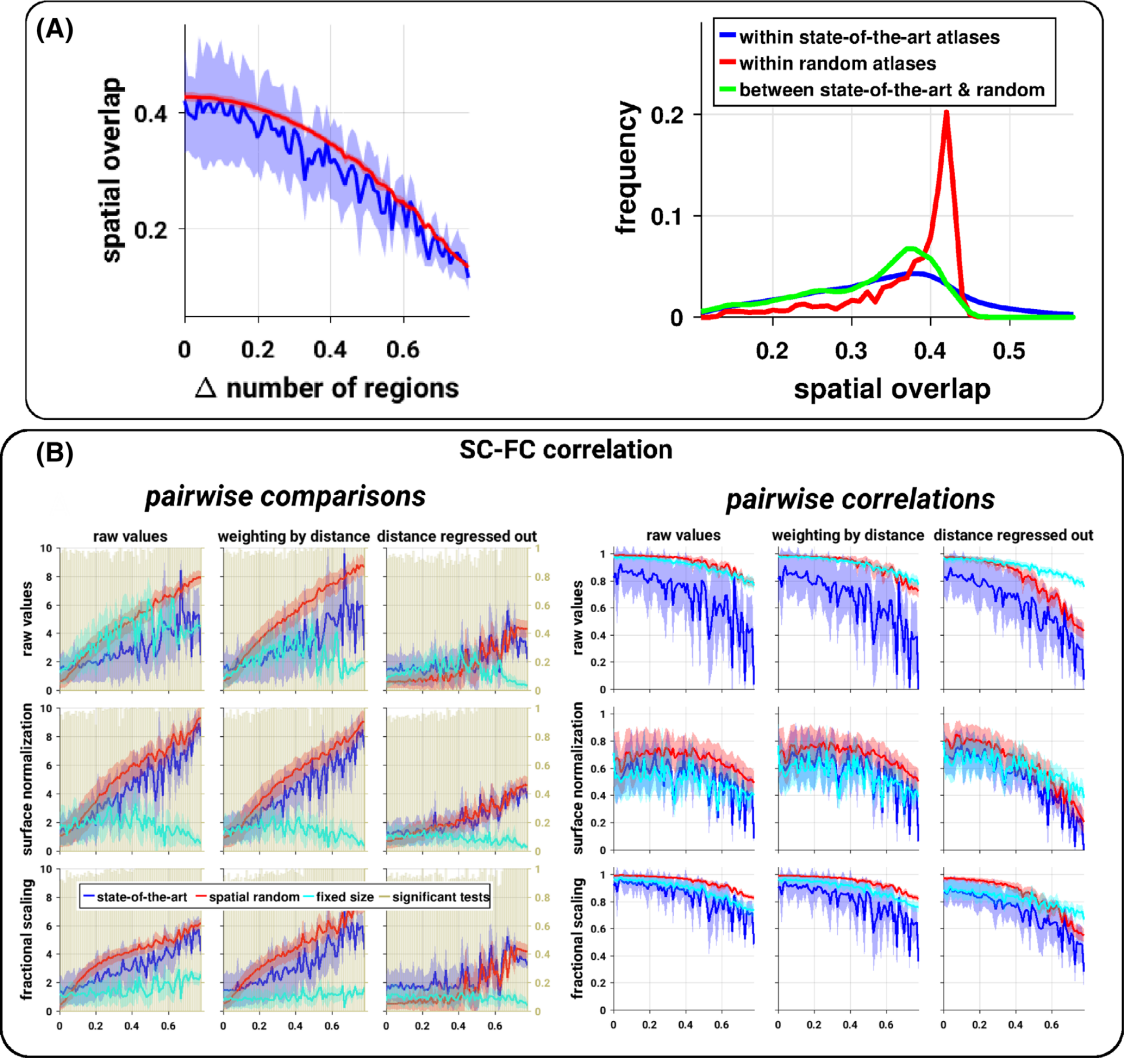
Brain SC–FC relationship, pairwise comparisons. (a) Pairwise spatial overlap between atlases. The adjusted Rand index values between atlases as a function of the normalized absolute difference of the number of regions (left plot) or their histograms (right plot). (b) Pairwise differences between SC–FC correlations, the Cohen's effect size of the difference (left) and the correlation coefficient (right) of the SC–FC correlation values between atlases according to various SC normalizations (subplots) as a function of the normalized absolute difference of the number of regions. For each subplot, the blue, red, and cyan curves represent the mean and *SD* (shaded area) across pairs of atlases of the same difference in size of the values from the comparison between SC–FC relationship values of the state‐of‐the‐art atlases, spatially random parcellations and fixed size parcellations, respectively. The dark beige histograms represent the proportion of pairs of state‐of‐the‐art atlases at a given normalized absolute difference for which there is a significant statistical difference (paired t‐tests corrected, *p* < .01)

We then asked how the atlases differ to one another in terms of spatial organization. The spatial overlap between atlases was relatively high, with decreasing similarity when the difference between the number of regions increased (Figure 4). Beyond the overall patterns of SC–FC relationship, we also assessed whether there exist significant differences across state‐of‐the‐art atlases. We observed a large number (more than 90%) of significant differences in the SC–FC correlation between atlases (Figure 4), while we observed only a moderate number of statistical differences (about 50%) for the overlap between SC and FC partitions (Figure S2). In general, differences in the SC–FC relationship increased with increasing difference in spatial scale, especially for the SC–FC correlation. Pairwise differences from the random parcellations followed a similar trend, while the differences from the fixed size parcellations were moderate and did not depend on the number of regions.

Additionally, the inter‐individual variability of the SC–FC relationship measures was generally highly correlated between atlases, with stronger covariations for atlases of similar size and for the SC–FC correlation compared to the partitions overlap. We also observed that the covariations were stronger between random parcels than between state‐of‐the‐art atlases (Figures 4 and S2).

### 3.5 Sensitivity to distance and preprocessing strategies

In general, spatially proximal regions are more likely to be strongly connected both structurally and functionally. In order to explore how much of the SC–FC relationship may be explained by distance, we report here the results when correcting for the distance. The weighting correction scheme only marginally altered the results, while the regression of the distance had a pronounced effect. Thus, we will focus only on the later. We observed a general drop in the SC–FC relationship values and a marked decrease as a function of the number of regions. As such, some of the SC–FC correlations were no longer significantly different from random expectations (permutation‐based tests corrected). Distance correction virtually abolished the SC–FC fingerprint where only a very small portion of values remained significant (permutation‐based tests corrected), see Figure 1. Interestingly, the distance correction renders a large number of the SC–FC correlations significantly higher than the ones obtained from the random parcels (Figure 2). Distance correction had no effect on the overlap between SC and FC partitions. Distance correction tended to slightly reduce the reliability of SC estimates (Figure 3). In terms of pairwise comparison, distance correction reduced the differences but they remained largely significant, while the covariations across atlases remained virtually unchanged (Figures 4 and S2).

When exploring the influence of alternative preprocessing strategies, the results remained overall consistent across the SC normalizations. However, we observed a negative impact of the widely employed surface normalization which expresses SC values per unit surface. SC–FC correlation dropped quickly to values close to zero when the number of regions increases, but the values remain significantly different from random expectations (permutation‐based tests corrected). In terms of SC–FC fingerprint, the surface normalization drop values to near zero regardless of the number of regions, hence making them largely nonsignificant (permutation‐based tests corrected). Using the surface normalization makes the SC–FC correlation virtually no longer significantly different from the random parcellations. Interestingly, the fractional scaling approach slightly increased the SC–FC correlation and fingerprint, and also increased positive differences against random parcels (Figures 1 and 2). Moreover, the fractional scaling tended to reduce the effect of the number of regions on the SC–FC correlation, which is corroborated with a higher SC reliability (Figure 3). The pairwise differences between atlases were not strongly affected by the SC normalizations; and covariations between atlases were reduced when using the surface normalization, while they were slightly higher when using the fractional scaling (Figure 4). The overlap between SC and FC partitions remains unchanged.

### 3.6 Is the Pearson correlation suitable?

So far, we have observed that the correlation between SC and FC is mainly influenced by the reliability of SC estimates, which decreased with an increasing number of areas. Such an effect can be potentially attributed to the skewed nature of the SC distributions (Figure S3), where the variability of the SC estimates may distort the resulting Pearson correlation values in a nontrivial way similar to the presence of outliers (Bishara & Hittner, 2015). In order to investigate such possibility, we computed nonparametric correlation coefficients using Spearman. Consequently, SC reliability increased significantly to a level similar to the ones from FC (Figure 3).

**Figure 5:**
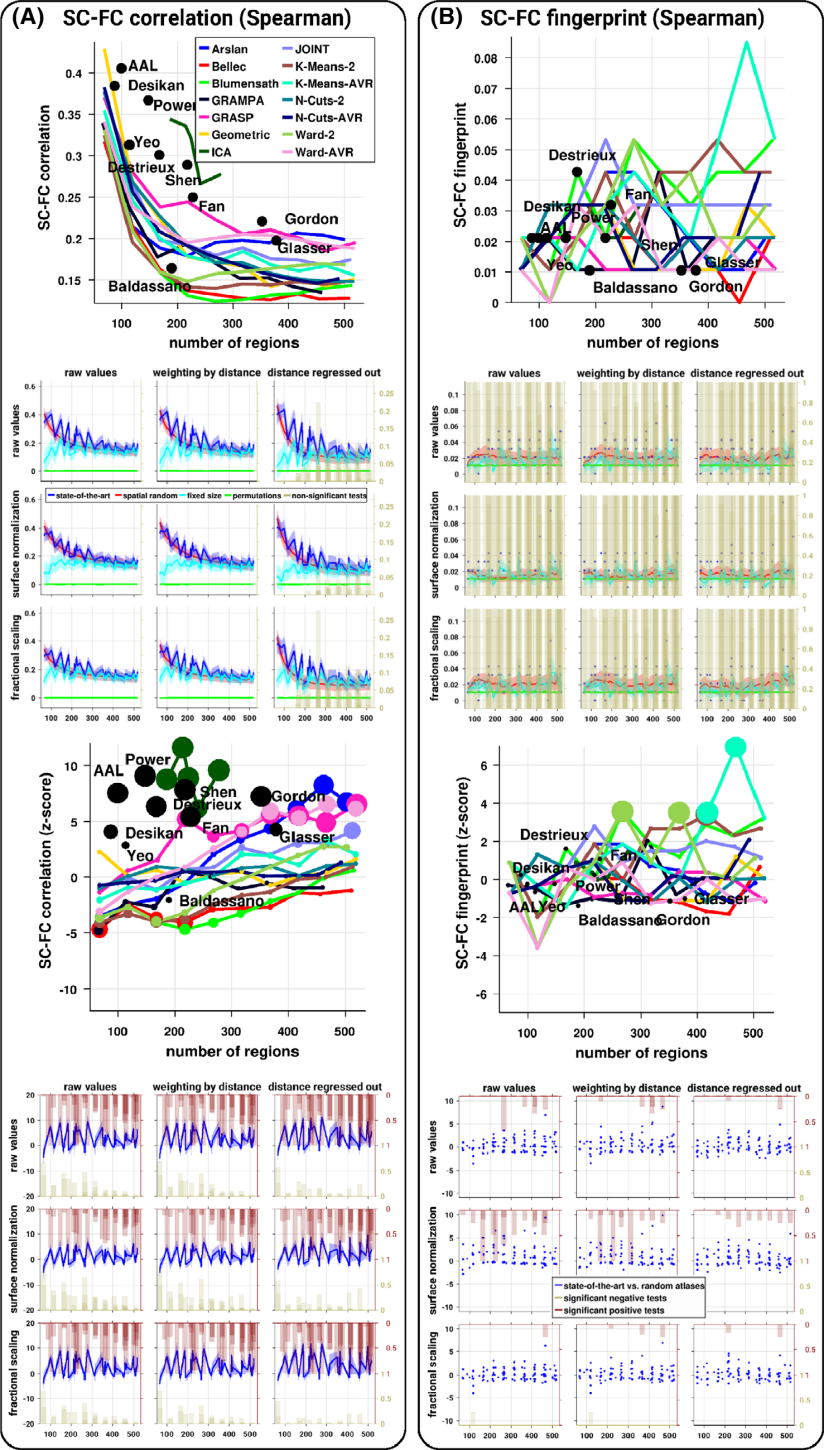
Brain SC–FC relationship across atlases when using the Spearman correlation. (a) SC–FC correlation, that is, the correlation between structural and functional connectivity patterns, computed for each subject and each atlas. (b) SC–FC fingerprint, that is, the proportion of subjects correctly identified according to their SC–FC correlation. Across the panels, the first row represents the average values per atlas as a function of the number of regions, the second row represents the SC–FC relationship according to various SC normalizations (subplots) as a function of the number of regions. For each subplot, the blue, red, cyan, and green curves represent the mean and *SD* (shaded area) across subjects and atlases of the same size of the SC–FC relationship from the state‐of‐the‐art atlases, spatially random parcellations, fixed size parcellations, and permutations, respectively. The dark beige histograms represent the proportion of subjects (or the proportion of atlases for the SC–FC fingerprint) at a given number of regions for whom there is no significant statistical difference compared to random expectations (permutation‐based tests corrected, *p* < .01). The third row represents the average normalized values per atlas as a function of the number of regions where the size of the points is proportional to the number of subjects significantly different from the random parcellations, the last row represents the normalized SC–FC relationship according to various SC normalizations (subplots) as a function of the number of regions. For each subplot, the blue curve represents the mean and *SD* (shaded area) of the normalized SC–FC relationship values across subjects and atlases of the same size of the state‐of‐the‐art atlases against random parcellations. The dark beige (resp. red) histograms represent the proportion of subjects (or the proportion of atlases for the SC–FC fingerprint) at a given number of regions for whom there is a significant statistical negative (resp. positive) deviation from the random parcellations (*z*‐tests corrected, *p* < .01). The blue dots in the SC–FC fingerprint subplots represent the state‐of‐the‐art atlases

We observed that the results remained largely consistent when computing the SC–FC correlation based on Spearman, with slightly higher values compared to Pearson (Figures 5 and S4). Moreover, a large number of the state‐of‐the‐art atlases were significantly superior (in terms of SC–FC correlation) compared to spatially random parcels. Nevertheless, the SC–FC fingerprint appeared completely abolished. These results demonstrated that the dependence on the number of regions in the correlation between SC and FC cannot be entirely attributed to the connectivity reliability.

Regressing out plausible spurious sources of variance in rfMRI data before computing FC did not alter the results when using Pearson (Figures S5 and S6) or Spearman (Figures S7 and S8). We only observed that the SC–FC correlation from the state‐of‐the‐art atlases were no longer significantly higher than that of random parcellations (Figures S5 and S7).

## 4 DISCUSSION

In the present study, we performed a comparison of a set of state‐of‐the‐art group‐wise brain atlases, to relate structural and functional connectivity derived from MRI data. We found that (a) there is a significant effect of brain parcellations on the SC–FC correlation and fingerprint, which is mainly driven by the number of areas; (b) the overlap between SC and FC partitions is virtually not influenced by the choice of the atlas nor by any normalization; (c) there are mixed differences in the SC–FC relationship when compared to purely random parcellations; (d) the influence of the number of areas cannot be attributed solely to the reliability of the connectivity estimates; (e) and beyond the influence of the number of regions, the spatial embedding of the brain (distance effect) can explain a large portion of the observed relationships. To the best of our knowledge, this is the first study that explores extensively the relationship between SC and FC across multiple factors, including not only the choice of the brain parcellation, but also commonly used preprocessing strategies.

A few studies have explored the influence of brain parcellations on the resulting structure and topology of whole‐brain structural (Bassett, Brown, Deshpande, Carlson, & Grafton, 2011; Zalesky et al., 2010) and functional (Arslan et al., 2018; Fornito, Zalesky, & Bullmore, 2010; Wang et al., 2009) connectivity networks, see de Reus & van den Heuvel, 2013 for review. Overall, although organizational principles appear preserved, these studies showed pronounced quantitative effects of the number of regions on several topological properties (including density, smallworldness, and scalefreeness), but rather consistent estimates at a given spatial scale (Bassett et al., 2011; Fornito et al., 2010; Wang et al., 2009; Zalesky et al., 2010). More recently, Arslan et al., 2018 did an extensive and systematic comparison of state‐of‐the‐art brain parcellations (where part of them were used here) on the resulting FC patterns using a variety of evaluation criteria including reproducibility, fidelity, agreement with alternative modalities and topological properties. Interestingly, across all criteria investigated, the brain atlases can hardly be distinguished from each other. The authors concluded that “*The results […] suggest that there is no optimal method able to address all the challenges faced in this endeavor simultaneously*” (Arslan et al., 2018). Here, we have observed very similar results with a pronounced effect of the number of regions, and specific behavior depending on the measure investigated. Moreover, there is no marked emerging trend when the state‐of‐the‐art atlases are compared to purely random parcellations, except when distance effect is corrected and/or Spearman is used, in which cases we show that most of the proposed atlases have a SC–FC correlation above the random parcels. SC–FC fingerprint appears to be mostly driven by the distance dependence on both measures of connectivity. We also report that the differences between atlases albeit small were systematic and lead to significant differences.

It is unclear which mechanisms lead to the dependence of the SC–FC relationship on the spatial scale. It is possible that with increasing number of regions the inter‐individual variability and hence the specificity of individuals increase in such a way that group‐wise atlases may not be any longer appropriate. Furthermore, the brain connectivity estimates at large scale become less robust, where the noise may play a more influential role. Here we show that FC is highly reliable even when it is subsampled at low rate, while the reliability of structural connectivity is mostly influenced by the choice of the measure of reliability itself. Shared variance in SC estimates between the state‐of‐the‐art atlases and the fixed‐size atlases is overall greater than 60% using Spearman, and it can be as low as 1% with Pearson. However, the pattern of SC–FC correlation remains largely unchanged using either one of the measure. Thus, the effect of the spatial scale cannot be explained only by the connectivity reliability.

Furthermore, tractography's ability to recover tracts is expected to decrease as a function of the distance due to technical biases. Consequently, structural connectivity estimates from diffusion MRI tractography are highly related to the fiber lengths (Roberts et al., 2017). Furthermore, distance is also a natural by‐product of the spatial and material (metabolic cost) constraints in the brain, making it a biological principle for the preferential connection of brain areas (Bullmore & Sporns, 2012). As such, it remains challenging to disentangle these two factors from tractography outputs. That is why, we explored the SC–FC relationship both with and without correcting such covariate to highlight its prominent effect.

Altogether, the present results and the abundance of available parcellations highlight the challenge of delineating brain areas, which render the concept of brain parcels somehow elusive (Sporns, 2013; Van Essen, Glasser, Dierker, Harwell, & Coalson, 2012). It has been sparsely but robustly documented that some anatomical brain connections have nonuniform spatial arrangements, for example, with the presence of gradients, which cannot be easily subdivided into areas. Connection topography, which consists of considering brain connectivity as a continuous field, has been proposed as an alternative way to study brain connectivity, complementary to the classic concept of network (Huntenburg, Bazin, & Margulies, 2018; Jbabdi, Sotiropoulos, & Behrens, 2013). It would be of considerable interest to explore the possibility to translate the concept of connectivity‐based structure–function relationship into such framework, all the more that connection topography appears to be a powerful approach to highlight inter‐individual variability (Tavor et al., 2016).

The present results are subject to several important methodological considerations. First and foremost, despite a concept being widely adopted, brain connectivity (both structural and functional) is under massive investigations and debates on the best approaches for obtaining proper estimates and on their interpretations. Structural connectivity can only be indirectly probed in vivo using dMRI together with computational tractography. While tractography‐based SC estimates correlate with tract‐tracing data (Calabrese, Badea, Cofer, Qi, & Johnson, 2015; Delettre et al., 2019; Donahue et al., 2016), it has also been shown that tractography faces a number of biases which result to a significant number of false positives (Jbabdi & Johansen‐Berg, 2011; Jones, Knösche, & Turner, 2013; Maier‐Hein et al., 2017; Reveley et al., 2015; Thomas et al., 2014). Thus, analyses using current dMRI‐based SC estimates, while being a powerful tool, should be interpreted with caution. On the other hand, FC remains an elusive concept given its vague definition and a lack of clear interpretations (Marrelec, Messé, Giron, & Rudrauf, 2016). We here employed the classic, basic and most widely used measure of Pearson correlation as a proxy. While there is an abundant literature which has provided meaningful insights into brain architecture using such measure, it would be informative to see whether alternative definitions nuance the SC–FC relationship. Whereas the SC–FC relationship was explored here at the network‐level, a potential new avenue would be to refine the analysis at the area‐level, where some specificities have been recently shown (Vázquez‐Rodríguez et al., 2019). Last but not least, we have here conducted an analysis on group‐wise atlases, while personalized parcellations remain only marginally investigated (Gratton et al., 2018). It would be interesting to explore whether subject‐specific atlases improve the correspondence between structure and function.

To conclude, the relationship between structural and functional connectivity depends on a myriad of factors. While the distinction between state‐of‐the‐art atlases and random parcellations is subtle in terms of the SC–FC measures, the choice of one over the other is most likely a matter of convenience. On one side, some state‐of‐the‐art atlases have the advantage of having interpretable local delineations. On the other side, random parcellations are easily implementable and adjustable, allowing to investigate the robustness of the results by statistical inference over multiple realizations and spatial scales. Structure–function analyses must balance the reliability of connectivity measures and the robustness of the association (the correspondence between SC and FC must be reasonably high), as such, atlases with 200–300 regions appear as a good compromise. The use of the fractional scaling to normalize SC values and the Spearman correlation to quantify associations are efficient steps for improving reliability. Furthermore, connectivity analyses should be done both with and without distance correction in order to probe the amount of variance explained by such prominent biological factor.

## CONFLICT OF INTEREST

The author declared no potential conflict of interest with respect to the research, authorship, and/or publication of this article.

## Supporting information


**Appendix S1**: Supporting Information.

## Data Availability

The data that support the findings of this study are available from the corresponding author upon reasonable request.

## ENDNOTES

1 The overlap between the 94 subjects used in the present study with those used in Arslan et al. (2018) (the 100 unrelated subjects) is low, only nine subjects are common to both datasets. The number of levels per atlas is 10, except for 2 atlases with only 5 and 9 levels, so we have in total 10 + 12 × 10 + 5 + 9 = 144.

2 Of note that the measure of reliability, as defined here, is biased towards high resolution atlases (with a high number of regions), as their average regions size gets close to the value sets for fixed‐size parcellations.
