# Supplementary material for: Parcellation influence on the connectivity‐based structure–function relationship in the human brain
Source: Hum Brain Mapp. 2019 Nov 19;41(5):1167–80. doi: 10.1002/hbm.24866 (PMC7267927; doi:10.1002/hbm.24866)
Supplement: Supplementary file 1 — Appendix S1: Supporting Information. [file HBM-41-1167-s001.pdf]

Supporting information for: Parcellation influence on the connectivity-based structure-function relationship in the human brain

Arnaud Messé

Department of Computational Neuroscience, University Medical Center Eppendorf, Hamburg University, Hamburg, Germany

Correspondence a.messe@uke.de

October 29, 2019

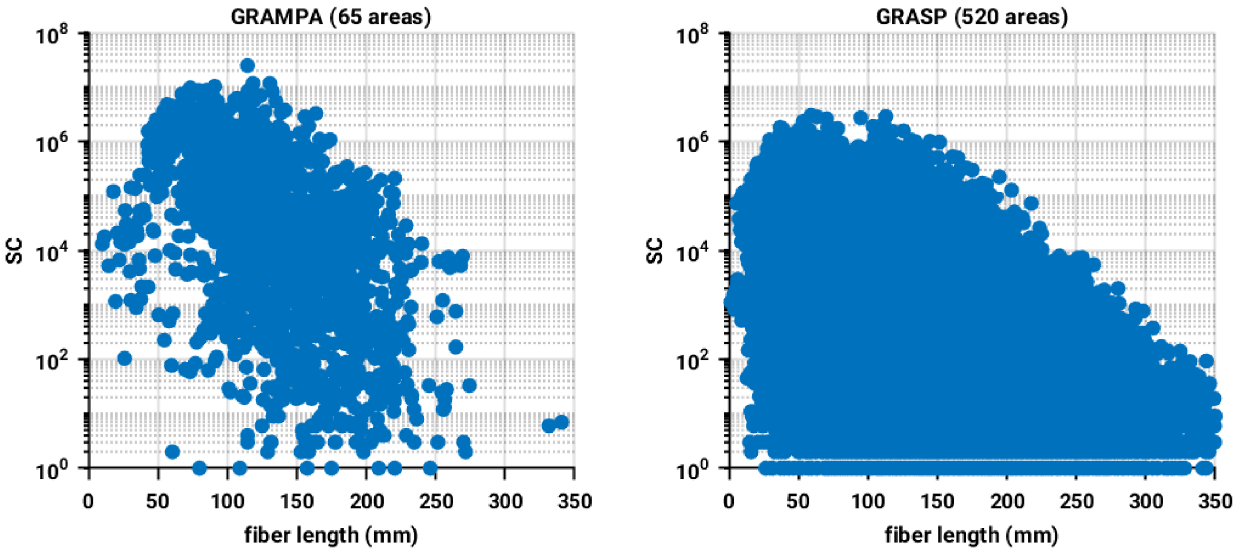

Figure S1: **Relationship between structural connectivity and fiber length.** Raw structural connectivity values as a function of the fiber lengths for an arbitrary subject and the atlases with the lowest (left) and highest (right) number of areas.

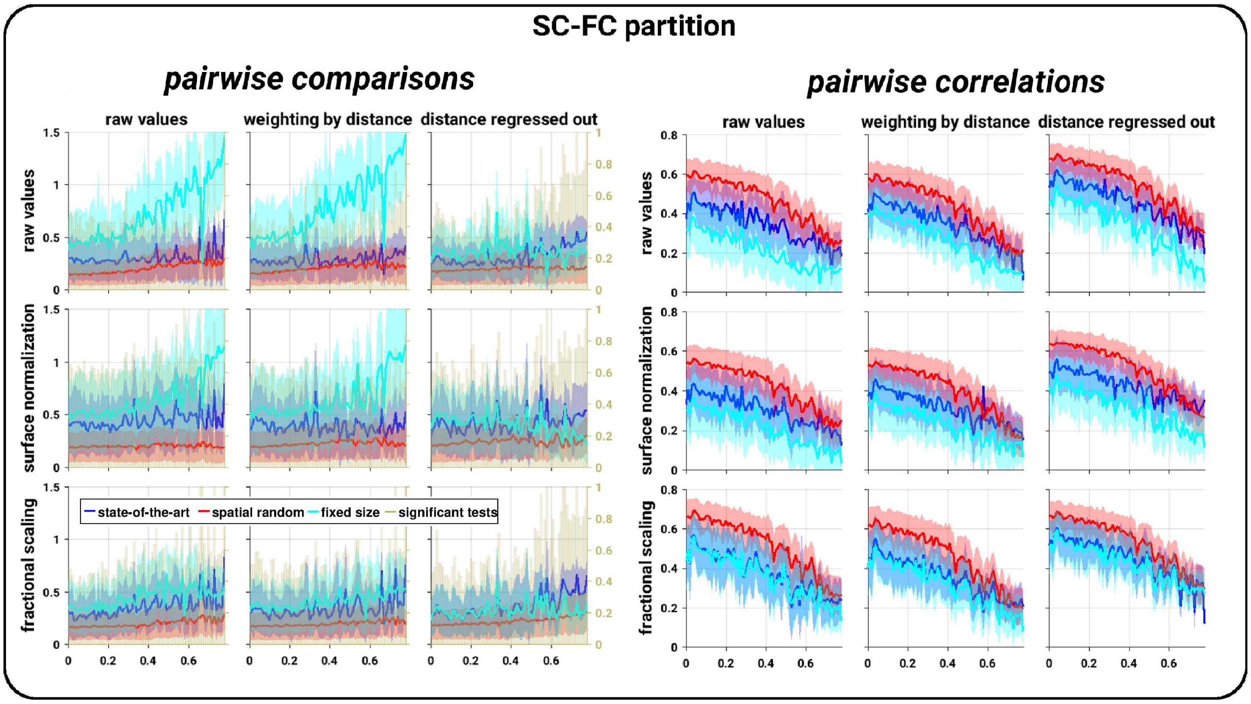

Figure S2: **Brain SC-FC relationship, pairwise comparisons.** Pairwise differences between SC-FC partitions' overlap, the Cohen's effect size of the difference (left) and the correlation coefficient (right) of the SC-FC partition between atlases according to various SC normalizations (subplots) as a function of the normalized absolute difference of the number of regions. For each subplot, the blue, red and cyan curves represent the mean and standard deviation (shaded area) across pairs of atlases of the same difference in size of the values from the comparison between SC-FC relationship values of the state-of-the-art atlases, spatially random parcellations and fixed size parcellations, respectively. The dark beige histograms represent the proportion of pairs of state-of-the-art atlases at a given normalized absolute difference for which there is a significant statistical difference (paired t-tests corrected, p<0.01).

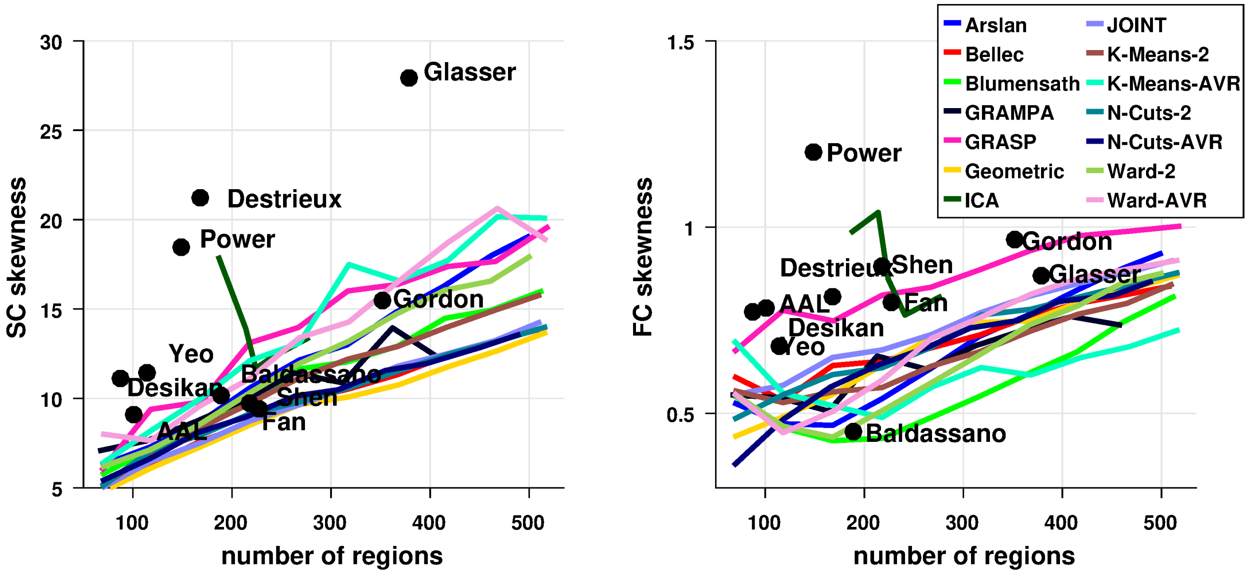

Figure S3: **Connectivity skewness.** The average skewness values of the structural (left) and functional connectivity matrices (right) per atlas as a function of the number of regions.

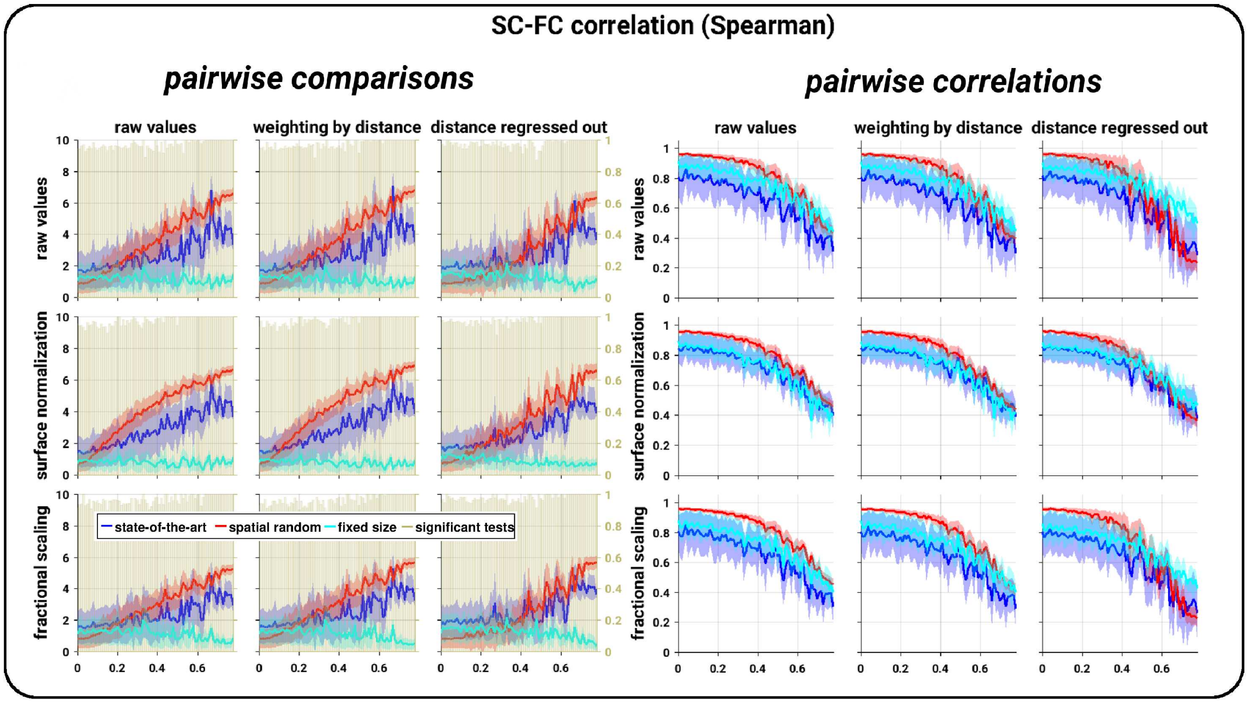

Figure S4: **Brain SC-FC relationship, pairwise comparisons when using the Spearman correlation.** Pairwise differences between SC-FC correlations, the Cohen's effect size of the difference (left) and the correlation coefficient (right) of the SC-FC correlation between atlases according to various SC normalizations (subplots) as a function of the normalized absolute difference of the number of regions. For each subplot, the blue, red and cyan curves represent the mean and standard deviation (shaded area) across pairs of atlases of the same difference in size of the values from the comparison between SC-FC relationship values of the state-of-the-art atlases, spatially random parcellations and fixed size parcellations, respectively. The dark beige histograms represent the proportion of pairs of state-of-the-art atlases at a given normalized absolute difference for which there is a significant statistical difference (paired t-tests corrected, p<0.01).

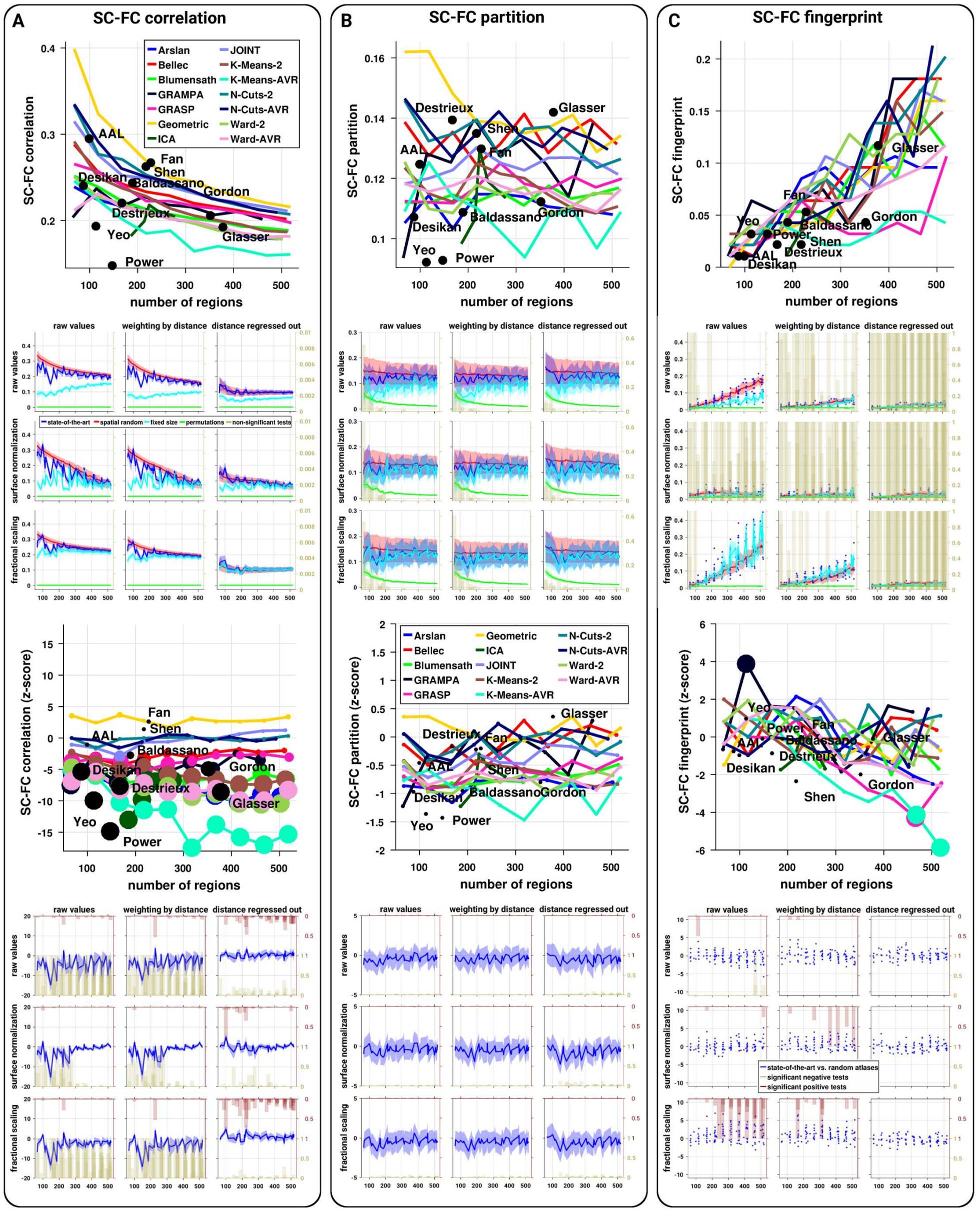

Figure S5: **Brain SC-FC relationship across atlases when FC is corrected for spurious variance.** (A) SC-FC correlation, ie., the correlation between structural and functional connectivity patterns, computed for each subject and each atlas. (B) SC-FC partition, ie., the overlap between communities extracted from SC and those from FC, computed for each subject and each atlas. (C) SC-FC fingerprint, ie., the proportion of subjects correctly identified according to their SC-FC correlation. Across the panels, the first row represents an illustration of the SC-FC relationship' measure, the second row represents the average values per atlas as a function of the number of regions, the last row represents the SC-FC relationship according to various SC normalizations (subplots) as a function of the number of regions. For each subplot, the blue, red, cyan and green curves represent the mean and standard deviation (shaded area) across subjects and atlases of the same size of the SC-FC relationship from the state-of-the-art atlases, spatially random parcellations, fixed size parcellations and permutations, respectively. The dark beige histograms represent the proportion of subjects (or the proportion of atlases for the SC-FC fingerprint) at a given number of regions for whom there is no significant statistical difference compared to random expectations (permutation-based tests corrected, p<0.01). The third row represents the average normalized values per atlas as a function of the number of regions where the size of the points is proportional to the number of subjects significantly different from the random parcellations, the last row represents the normalized SC-FC relationship according to various SC normalizations (subplots) as a function of the number of regions. For each subplot, the blue curve represents the mean and standard deviation (shaded area) of the normalized SC-FC relationship values across subjects and atlases of the same size of the state-of-the-art atlases against random parcellations. The dark beige (resp. red) histograms represent the proportion of subjects (or the proportion of atlases for the SC-FC fingerprint) at a given number of regions for whom there is a significant statistical negative (resp. positive) deviation from the random parcellations (z-tests corrected, p<0.01). The blue dots in the SC-FC fingerprint subplots represent the state-of-the-art atlases.

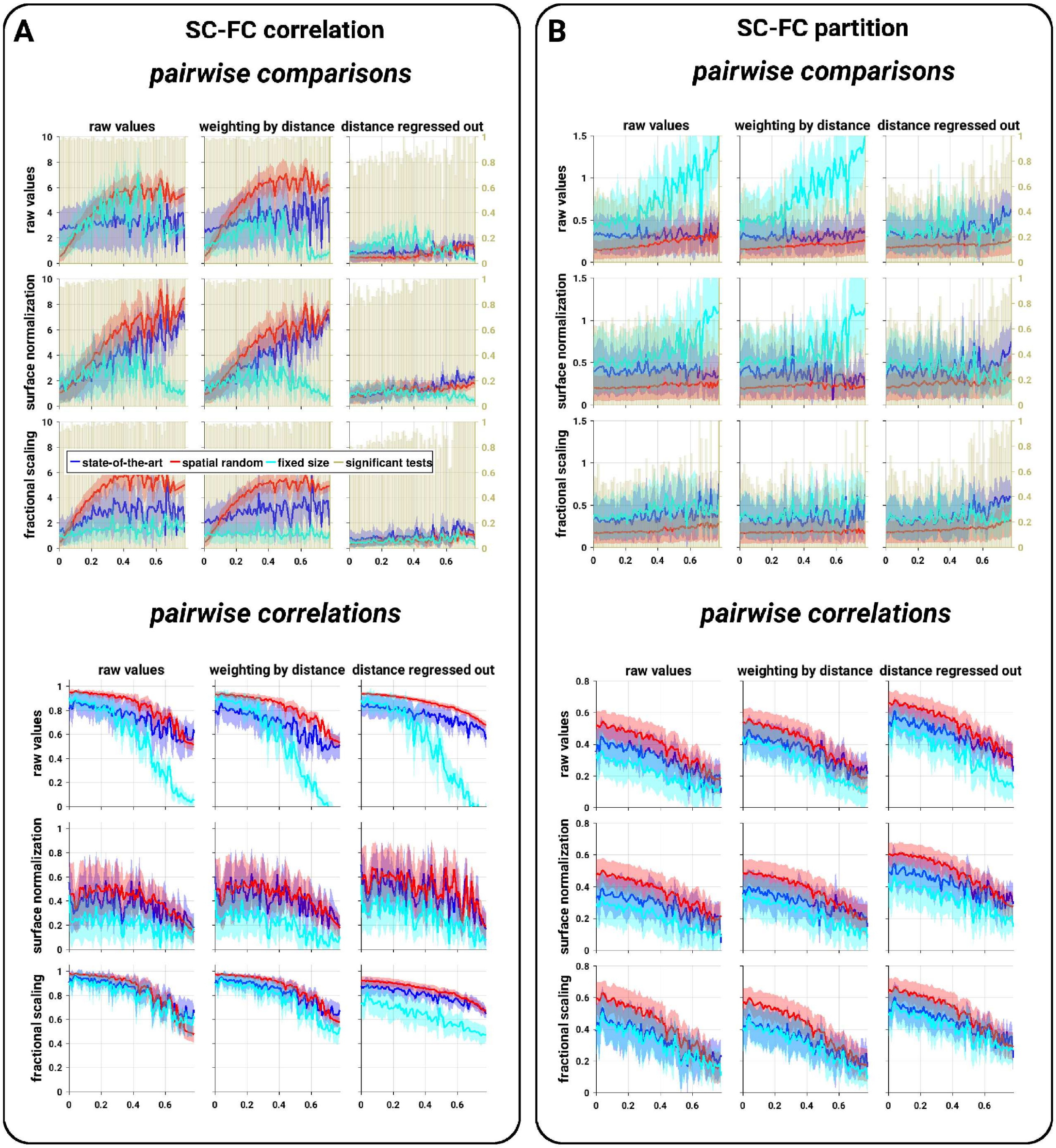

Figure S6: **Brain SC-FC relationship, pairwise comparisons when FC is corrected for spurious variance.** Pairwise differences between SC-FC correlations (A) and SC-FC overlaps (B). Across the panels, the first (resp. second) row represents the Cohen's effect size of the difference (resp. the correlation coefficient) of the SC-FC relationship between atlases according to various SC normalizations (subplots) as a function of the normalized absolute difference of the number of regions. For each subplot, the blue, red and cyan curves represent the mean and standard deviation (shaded area) across pairs of atlases of the same difference in size of the values from the comparison between SC-FC relationship values of the state-of-the-art atlases, spatially random parcellations and fixed size parcellations, respectively. The dark beige histograms represent the proportion of pairs of state-of-the-art atlases at a given normalized absolute difference for which there is a significant statistical difference (paired t-tests corrected, p<0.01).

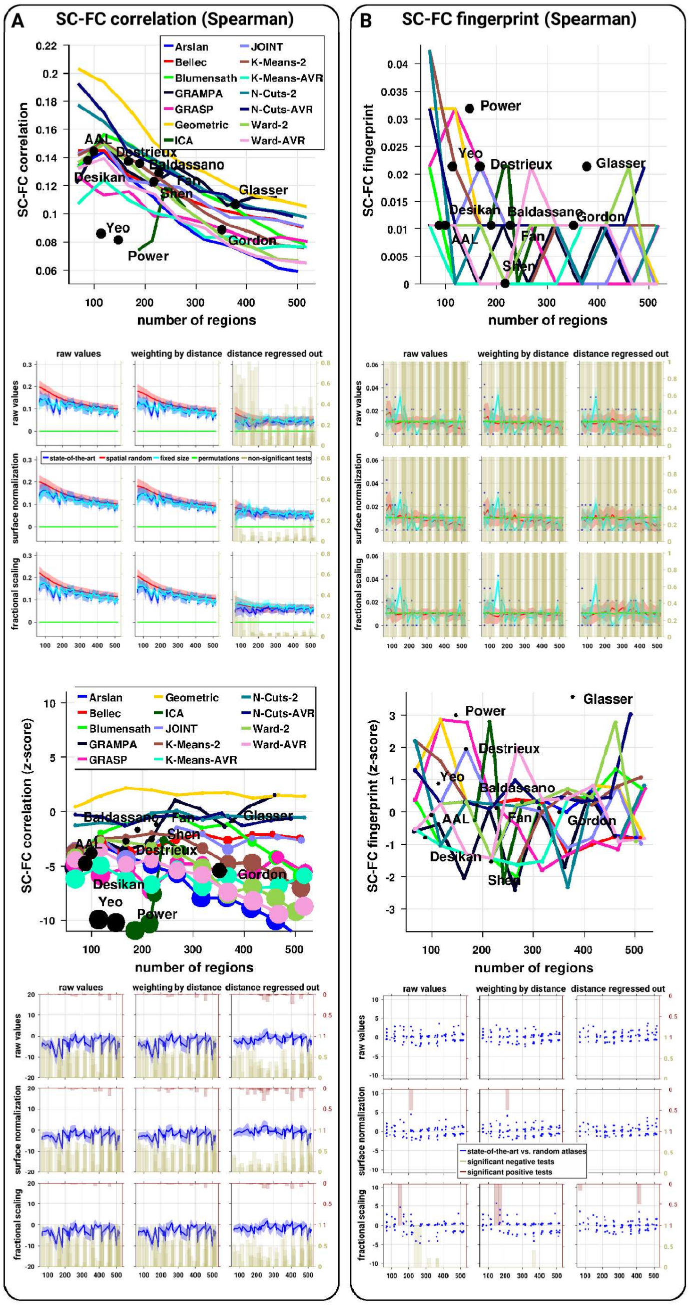

Figure S7: **Brain SC-FC relationship across atlases when FC is corrected for spurious variance and using the Spearman correlation.** (A) SC-FC correlation, ie., the correlation between structural and functional connectivity patterns, computed for each subject and each atlas. (B) SC-FC fingerprint, ie., the proportion of subjects correctly identified according to their SC-FC correlation. Across the panels, the first row represents represents the average values per atlas as a function of the number of regions, the second row represents the SC-FC relationship according to various SC normalizations (subplots) as a function of the number of regions. For each subplot, the blue, red, cyan and green curves represent the mean and standard deviation (shaded area) across subjects and atlases of the same size of the SC-FC relationship from the state-of-the-art atlases, spatially random parcellations, fixed size parcellations and permutations, respectively. The dark beige histograms represent the proportion of subjects (or the proportion of atlases for the SC-FC fingerprint) at a given number of regions for whom there is no significant statistical difference compared to random expectations (permutation-based tests corrected, p<0.01). The third row represents the average normalized values per atlas as a function of the number of regions where the size of the points is proportional to the number of subjects significantly different from the random parcellations, the last row represents the normalized SC-FC relationship according to various SC normalizations (subplots) as a function of the number of regions. For each subplot, the blue curve represents the mean and standard deviation (shaded area) of the normalized SC-FC relationship values across subjects and atlases of the same size of the state-of-the-art atlases against random parcellations. The dark beige (resp. red) histograms represent the proportion of subjects (or the proportion of atlases for the SC-FC fingerprint) at a given number of regions for whom there is a significant statistical negative (resp. positive) deviation from the random parcellations (z-tests corrected, p<0.01).

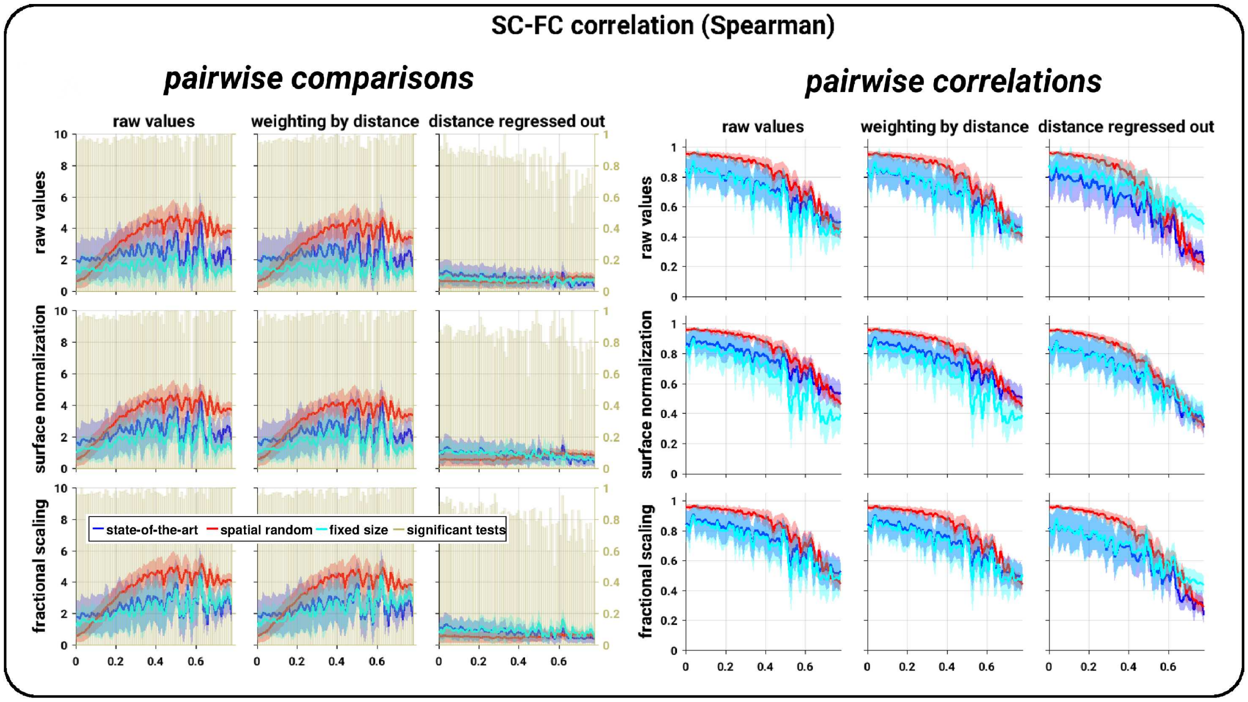

Figure S8: **Brain SC-FC relationship, pairwise comparisons when FC is corrected for spurious variance and using the Spearman correlation.** Pairwise differences between SC-FC correlations, the Cohen's effect size of the difference (left) and the correlation coefficient (right) of the SC-FC correlation between atlases according to various SC normalizations (subplots) as a function of the normalized absolute difference of the number of regions. For each subplot, the blue, red and cyan curves represent the mean and standard deviation (shaded area) across pairs of atlases of the same difference in size of the values from the comparison between SC-FC relationship values of the state-of-the-art atlases, spatially random parcellations and fixed size parcellations, respectively. The dark beige histograms represent the proportion of pairs of state-of-the-art atlases at a given normalized absolute difference for which there is a significant statistical difference (paired t-tests corrected, p<0.01).
